# Intraflagellar Transport (IFT) Protein IFT25 Is a Phosphoprotein Component of IFT Complex B and Physically Interacts with IFT27 in *Chlamydomonas*

**DOI:** 10.1371/journal.pone.0005384

**Published:** 2009-05-01

**Authors:** Zhaohui Wang, Zhen-Chuan Fan, Shana M. Williamson, Hongmin Qin

**Affiliations:** Department of Biology, Texas A&M University, College Station, Texas, United States of America; University of Hong Kong, Hong Kong

## Abstract

**Background:**

Intraflagellar transport (IFT) is the bidirectional movement of IFT particles between the cell body and the distal tip of a flagellum. Organized into complexes A and B, IFT particles are composed of at least 18 proteins. The function of IFT proteins in flagellar assembly has been extensively investigated. However, much less is known about the molecular mechanism of how IFT is regulated.

**Methodology/Principal Findings:**

We herein report the identification of a novel IFT particle protein, IFT25, in *Chlamydomonas*. Dephosphorylation assay revealed that IFT25 is a phosphoprotein. Biochemical analysis of temperature sensitive IFT mutants indicated that IFT25 is an IFT complex B subunit. *In vitro* binding assay confirmed that IFT25 binds to IFT27, a Rab-like small GTPase component of the IFT complex B. Immunofluorescence staining showed that IFT25 has a punctuate flagellar distribution as expected for an IFT protein, but displays a unique distribution pattern at the flagellar base. IFT25 co-localizes with IFT27 at the distal-most portion of basal bodies, probably the transition zones, and concentrates in the basal body region by partially overlapping with other IFT complex B subunits, such as IFT46. Sucrose density gradient centrifugation analysis demonstrated that, in flagella, the majority of IFT27 and IFT25 including both phosphorylated and non-phosphorylated forms are cosedimented with other complex B subunits in the 16S fractions. In contrast, in cell body, only a fraction of IFT25 and IFT27 is integrated into the preassembled complex B, and IFT25 detected in complex B is preferentially phosphorylated.

**Conclusion/Significance:**

IFT25 is a phosphoprotein component of IFT particle complex B. IFT25 directly interacts with IFT27, and these two proteins likely form a subcomplex *in vivo*. We postulate that the association and disassociation between the subcomplex of IFT25 and IFT27 and complex B might be involved in the regulation of IFT.

## Introduction

Cilia and flagella are microtubule-based appendages extending from the basal body of almost all eukaryotic cells, and are classified as either motile or primary [1]. Motile cilia or flagella such as *Chlamydomonas* flagella, sperm flagella and respiratory tract epithelial cell cilia are responsible for movement or generation of fluid flow. In contrast, primary cilia are non-motile organelles that are critically involved in visual, olfactory and auditory signal transduction and play key roles in regulation of gene expression, development and behavior [2].

As evolutionally conserved cellular appendages, cilia and flagella are assembled and maintained by a motility process called intraflagellar transport (IFT). IFT was first discovered in the unicellular green algae *Chlamydomonas* [1] and refers to the rapid, bidirectional movement of multimeric protein particles termed IFT particles along the axoneme [3]. IFT machinery includes three key components: the IFT particle, the anterograde motors and the retrograde motor [1]. IFT particles are transported from the basal body to the flagellar tip by the coordinating action of either the heterotrimetric kinesin-II motor alone or kinesin-II together with the homodimer OSM-3/KIF17 motor, depending on specific organisms [4], [5], [6]. In the reverse direction, this movement is powered by the motor protein cytoplasmic dynein 1b [7], [8], [9], [10], [11]. The main constituents of IFT particles are organized into two protein complexes, A and B, which contain at least six or twelve polypeptides, respectively [5], [12], [13]. IFT particle proteins are thought to serve as scaffolds to facilitate the attachment of cargo proteins to IFT motors [14]. Thus the movement of IFT particles mediates the transport of flagellar precursors [14], [15], [16], membrane signaling proteins [17], [18], [19], and turnover products of the flagella [14], [20], [21], [22], [23] into or out of the flagellar compartment. For example, IFT particle complex B subunit IFT46 can bind to ODA16 directly, and thus serves as a bridge between IFT particles and outer dynein for efficient dynein transport into the flagellar compartment [15]. Consequently, mutations occurring in the components of the IFT machinery affect ciliary or flagellar assembly, maintenance and function [1], [24], [25].

Although the critical roles of IFT in flagellar assembly have been well established, little is known about the regulation of IFT, such as the loading of flagellar precursors at the flagellar base and the release of the turnover products at the flagellar tip. In particular, the molecular mechanism of how anterograde IFT is initiated is problematic to address experimentally. This is largely due to the fact that anterograde IFT, once impaired or disrupted, prevents flagellar assembly. This situation makes it difficult to differentiate the molecules that are solely involved in the activation of anterograde IFT but not other aspects of the flagella assembly. To date, a strong correlation between IFT complex A subunits and retrograde IFT has been observed. In *Chlamydomonas*, the defects leading to the disappearance of complex A subunits cause impaired retrograde IFT but not anterograde IFT, and thus result in accumulation of proteins such as complex B subunits at the flagellar tip [26], [27], [28]. Preferential accumulation of complex B subunits at the flagellar tip was also observed in a complex A mutant *ift122a* of *Tetrahymena* [29], further emphasizing the importance of complex A subunits in retrograde IFT. Additionally, evidence has showed that the IFT complex B subunit is involved in retrograde IFT as well. An *ift172* mutant of *Tetrahymena* encoding a C-terminal truncated IFT172 displays accumulation of not only the truncated IFT172 itself, but also other IFT particle proteins at the flagellar tip [30]. This phenotype is recapitulated in *Chlamydomonas*; a point mutation in the IFT172 gene causes accumulation of IFT particle proteins at the flagellar tip [31]. In summary, all data clearly have demonstrated that both complex A and B subunits are involved in the regulation of retrograde IFT.

In this study, we report the identification of a novel IFT complex B subunit, IFT25, and the exploration of its possible regulatory role in IFT. The mouse homologue of IFT25 has been recently identified to interact with complex B [32]. In *Chlamydomonas*, IFT25 is a phosphoprotein, which is detectable in both flagella and cell body compartments. We have also determined its cellular distribution pattern and investigated its interaction with another IFT complex B subunit, IFT27. IFT27 is a small Rab-like GTPase component of IFT complex B [33]. In *Chlamydomonas*, IFT27 is unique among the IFT particle proteins in that this protein is not only essential for ciliogenesis, but is also involved in the control of cell cycle progression and cytokinesis [33]. The human homologue of IFT25 has been shown to interact with IFT27 physically [34], thus indicating that IFT25 probably pursues its function by binding to IFT27. From this study, we have attempted to gain a better understanding of how IFT25 is involved in ciliogenesis and its relationship with IFT27 during this process.

## Results

### Identification of IFT25: a phosphoprotein component of IFT particle

**Figure 1:**
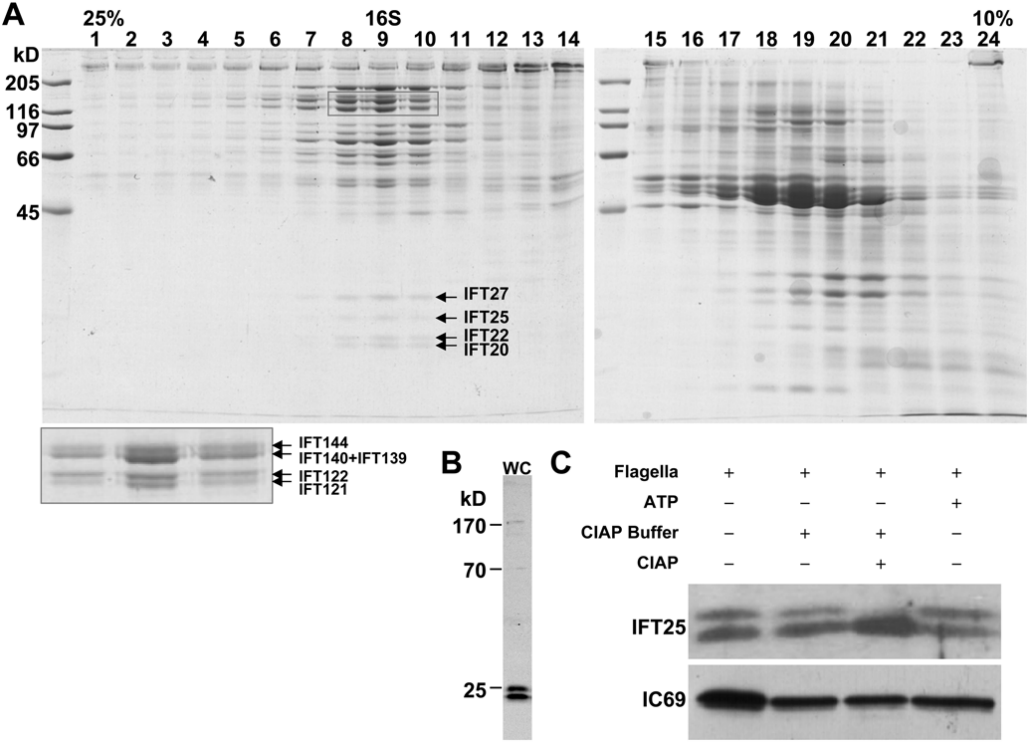
IFT25 is a phosphoprotein component of the IFT particle. (A). Identification and purification of IFT25 from isolated flagella extracts of *Chlamydomonas*. The soluble flagellar proteins isolated from flagella of the wt cells were fractionated on a 12-ml 10–25% sucrose gradient. The Coomassie blue-stained 10% SDS-PAGE gel of gradient fractions 1 (25% sucrose) to 24 (10% sucrose) is shown. IFT particle proteins peaked at the 16S fractions. The proteins highlighted in grey-lined rectangles are IFT particle complex A proteins with high molecular weights. IFT27, IFT25, and IFT22 polypeptides are highlighted with arrows. (B). Antibody α-IFT25 recognizes two bands on an immunoblot of wt whole cell extracts. (C). IFT25 is a phosphoprotein. Two bands were detected from the flagella extracts using an antibody against IFT25 protein on Western blots. After the treatment with calf intestine alkaline phosphatase (CIAP), the upper band disappeared and the intensity of the lower band was correspondingly increased. Addition of exogenous ATP elevated the level of the upper band, and the lower band showed a corresponding decreased intensity. The axonemal protein IC69 was used as a loading control.

Using an improved purification method (see the materials and methods section below), we were able to identify three novel IFT particle proteins from the flagellar 16S sucrose gradient fractions of *Chlamydomonas*. These proteins included IFT121, IFT25 and IFT22 (Fig. 1A), which were named after their actual protein sizes on SDS-PAGE gel. To identify the gene encoding IFT25, the Coomassie blue-stained band corresponding to IFT25 was subjected to microsequence analysis by mass spectrometry. Two resultant peptide sequences including LQTEVVHQVNIR and VSVVGGDDDGGGYDEPGGGYGSMQR(Q) were obtained and used to search the *Chlamydomonas* EST database. The result revealed that both peptides are the internal sequences of a conserved flagellar protein called Flagellar Associated Protein FAP232 (http://genome.jgi-psf.org/cgi-bin/dispGeneModel?db=Chlre3&tid=98791). To determine the IFT25 cDNA sequence, IFT25 transcripts were reverse-transcribed, followed by PCR amplification, and the resultant RT-PCR products were sequenced. Our sequencing data showed that the 3′ end of FAP232 was predicted incorrectly in the database. The correct IFT25 cDNA is 570 nucleotides in length and encodes a protein with a predicted size of approximately 20 kD and pI of 4.69. The IFT25 cDNA sequence is available from GenBank/EMBL/DDBJ under accession no. EF593953.

**Figure 2:**
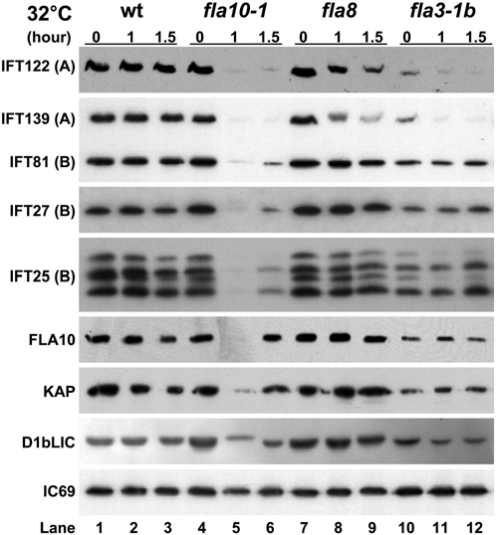
IFT25 behaves similarly to complex B subunits but not complex A subunits in anterograde IFT FLA10-kinesin-II *ts* mutants. Wild-type, *fla10-1*, *fla8*, and *fla3-1b* mutants grown in M1 medium at 18°C were incubated at 32°C for 0, 1.0 or 1.5 hours prior to deflagellation. The flagella were purified and analyzed with 10% SDS-PAGE and immunoblotting. The antibodies used in immunoblotting included those against IFT25, complex A subunits IFT139 and IFT122, complex B subunits IFT81 and IFT27, FLA10, KAP, and D1bLIC as indicated on the left. IC69 was used as a loading control.

**Figure 3:**
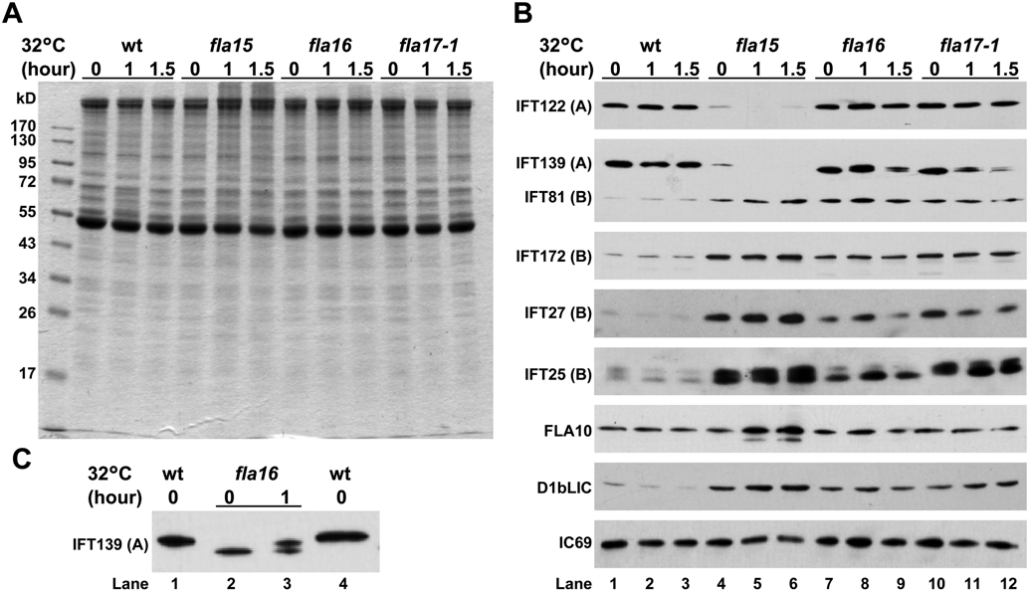
IFT25, similarly to IFT complex B subunits, accumulates in three retrograde IFT *ts* mutants. (A). Coomassie blue staining of the purified flagella proteins on a SDS-PAGE gel. Wild-type, *fla15*, *fla16*, and *fla17-1* mutants grown in M1 medium at 18°C were incubated at 32°C for 0, 1.0 or 1.5 hours prior to deflagellation. The flagellar proteins were purified, quantified, and separated on a 10% SDS-PAGE gel with a loading of the same amount of proteins per lane. The molecular markers are labeled on the left. The incubation time is marked on the top. (B). Immunoblots derived from the SDS-PAGE gel shown in A. Flagellar proteins on the gel were transferred to a nitrocellulose membrane. The membrane was then probed with antibodies against IFT25, the complex A subunits IFT122 and IFT139, the complex B subunits IFT172, IFT81, and IFT27, FLA10, or D1bLIC, and a flagellar axonemal protein IC69 as indicated on the left. Two bands corresponding to IFT139 were detected from the flagella of *fla16* and *fla17-1* mutants when incubated at 32°C. (C). Two different isoforms of IFT139 were detected. The immunoblot probed with the IFT139 antibody showed that the flagella isolated from *fla16* mutant contained a single IFT139 band migrating faster than that isolated from wt cells when incubated at 18°C. When the temperature was shifted to 32°C, a slower-migrating band with a size similar to IFT139 appeared.

To further characterize IFT25, a polyclonal antibody was raised against an internal peptide of IFT25 (see the materials and methods section below) and employed to detect the endogenous IFT25 on Western blots. More than one band with a size of approximately 25 kD was detected from either whole flagella extracts (Figs. 1C, 2, and 3B) or cell body lysates (Fig. 1B). As shown in Fig. 1C, the slower-migrating IFT25 band completely disappeared when the flagella extract was incubated with calf intestine alkaline phosphatase (CIAP), and the intensity of IFT25 band with faster migration was increased correspondingly. This result indicated that IFT25 is a phosphoprotein. Furthermore, addition of ATP to the flagella extracts resulted in a conversion of some IFT25 from its non-phosphorylated form to its phosphorylated form (Fig. 1C), indicating that IFT25 could be phosphorylated by a flagellar kinase. Considering that either two (Figs. 1B, IC, and 3B) or four (Fig. 2) bands were detected on Western blots, IFT25 must bear at least two phosphorylation sites in order to show three different phosphorylation states.

### The amount of IFT25 changes in a parallel manner with complex B subunits in the flagella of temperature sensitive IFT mutants

Temperature sensitive (*ts*) *Chlamydomonas* mutants *fla10-1*, *fla8*, and *fla3-1b* harbor point mutations in either of two heterotrimeric kinesin-II motor subunits, FLA10 [35] and FLA8 [36] or the non-motor KAP subunit, FLA3 [37]. These mutants are functionally normal in ciliogenesis at permissive temperature (18°C) but defective in anterograde IFT at non-permissive temperature (32°C). We measured the amount of change in IFT25 relative to other IFT particle proteins in the whole flagella of these three *ts* mutants. Similar to complex A subunits IFT139 and IFT122 and complex B subunits IFT81 and IFT27 that were included as controls (Fig. 2, lanes 5 and 6), the amount of IFT25 was significantly decreased in the flagella of the *fla10-1* mutant at 32°C. This result revealed that the entrance of IFT25 into flagella is FLA10-dependent. At the non-permissive temperature, the amounts of IFT25 and other complex B subunits IFT81 and IFT27 remained unchanged in the flagella of the *fla8* mutant, whereas slightly reduced amounts of complex A subunits IFT139 and IFT122 were observed (Fig. 2, lanes 8 and 9). In the flagella of the *fla3-1b* mutant incubated at either the permissive (18°C) or the non-permissive temperature, the amounts of both complexes A and B subunits were significantly decreased (Fig. 2, lanes 10, 11, and 12). In this case, IFT25 and all IFT complex B subunits were maintained at the same level at both temperatures. However, the amounts of complex A subunits dropped to an even lower level at the non-permissive temperature. In summary, these data showed that, although *fla8*, and *fla3-1b* mutants exhibited distinct effects on the amount change in IFT complex A and B subunits in flagella, the change in IFT25 paralleled to complex B subunits rather than complex A subunits.

Previous studies showed that IFT complex A subunits rather than complex B subunits decreased significantly in flagella when three retrograde IFT *ts* mutants *fla15*, *fla16*, *and fla17-1* [26], [27] are incubated at either the permissive or the non-permissive temperature. We measured the amount change in IFT25 as well as other components of IFT machinery, including complex A subunits IFT139 and IFT122 and complex B subunits IFT172, IFT81 and IFT27 in the whole flagella of these three *ts* mutants. It was obvious that the changes in complex A subunits were not consistent among the three mutants (Figs. 3A and 3B). At either temperature, IFT139 and IFT122 were virtually depleted from the flagella of the *fla15* mutant (Fig. 3B, lanes 4–6). In the flagella of the *fla16* and *fla17-*1 mutants, the amount of IFT122 was maintained at the level similar to wild-type (wt) at either temperature (Fig. 3B, lanes 7–12). In contrast, the amount of IFT139 was maintained at the near wt level at 18°C, but gradually dropped after the temperature was shifted to 32°C (Fig. 3B, lanes 7–12). In addition, it was interesting to note that instead of one band corresponding to IFT139, which was observed in wt flagella, two IFT139 bands were shown in the flagella of both the *fla16* and *fla17-1* mutants (Fig. 3B, lanes 9, 11, and 12). For the *fla16* mutant, it was clear that, at permissive temperature, a single band with a molecular weight smaller than IFT139 was detected (Fig. 3C, lane 2). When temperature was shifted to 32°C, one additional band with a size similar to IFT139 appeared (Fig. 3C, lane 3). In all three mutants, the amounts of flagellar complex B subunits and IFT25 were elevated significantly at both temperatures. The amount of flagellar IFT25 was increased in a parallel manner with complex B subunits rather than complex A subunits.

### IFT25 has a unique cellular distribution pattern and physically binds to IFT27

**Figure 4:**
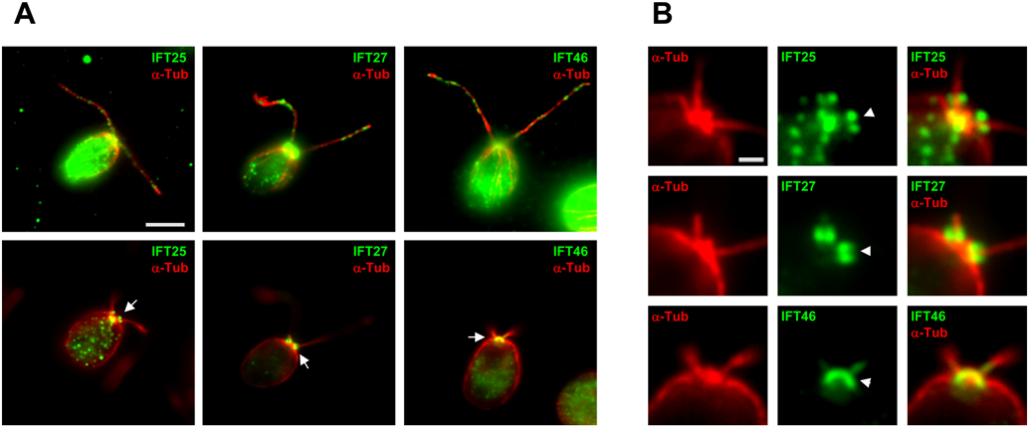
IFT25 has a unique distribution pattern. (A). Flagella and cell body distribution patterns of IFT25, IFT27, and IFT46. Fluorescent microscopy assay showed that IFT25, IFT27, and IFT46 (green) had spotted distribution along the entire length of flagella, a typical pattern of IFT proteins (A. top row). α-tubulin (red) was used to serve as a marker for basal bodies and flagella. The bottom row shows the cell body distributions of those three proteins. The scale bar is 5 µm.The details of staining at the basal bodies are enlarged and shown in B. (B). Cell body distribution patterns of IFT25, IFT27, and IFT46. Using IFT27 and IFT46 as controls, fluorescent microscopy assay showed that IFT25 (green) was localized into two regions: two bright dots comprising a region directly above each basal body, probably the transition zones (above the α-tubulin shown as red in B. top row) and a band possibly below each basal body (partially overlapped with the α-tubulin shown as red). IFT27 (green) was localized into regions that were probably the transition zones, but was not present in the proximal areas of the basal bodies (B. middle row). IFT46 (green) was found to overlap with α-tubulin (red) at the basal bodies (B. bottom row). The scale bar equals 1 µm.

It is known that IFT complex B subunits such as IFT46 and IFT81 [5], [14], [16], [26], [38] and subunits of cytoplasmic dynein 1b [7], [10], [11], [39] and Fla10-kinesin-II [5], [37], [38] all reside in the same region in basal bodies. In this study, immunofluorescent microscopy assay was applied to investigate the cellular distribution pattern of IFT25 together with complex B subunits IFT46 and IFT27 as controls. As shown in Fig. 4A, all three proteins showed a spotted distribution along the entire length of the flagella, which is typical for IFT proteins [16], [33]. This result is consistent with that IFT25 is an IFT particle protein. Unlike IFT46 that was concentrated in basal bodies, IFT27 was detected mainly in a region directly above the basal bodies, probably the transition zones (bottom row, Fig. 4A and Fig. 4B). Interestingly, IFT25 was detected at two different regions. It was clear that IFT25 and IFT27 overlapped as two bright dots comprising a region right above each basal body. At the basal body region, IFT25 had a distribution pattern similar to IFT46 as a band perpendicular to each basal body (bottom row, Fig. 4A and Fig. 4B) [5], [14], [16]. However, it was obvious that IFT25 was concentrated in a region directly below the marker protein á-tubulin, indicating that this protein did not overlap or only partially overlapped with IFT46. From Fig. 4B, the possibility that IFT25 and IFT27 could partially overlap with IFT46 at the distal end of basal bodies cannot be rejected. In summary, these results demonstrated that IFT25 has a unique distribution pattern among complex B subunits. IFT25 overlapped with IFT27 at regions around the transition zones and was present in basal bodies as well.

**Figure 5:**
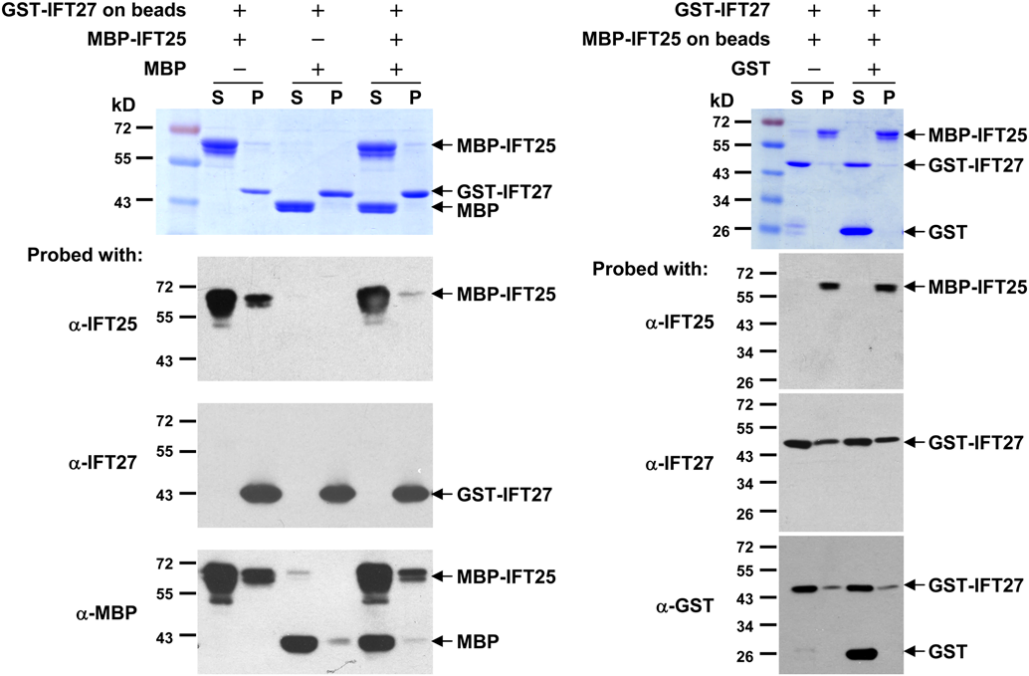
IFT25 interacts physically with IFT27. Purified GST-tagged IFT27 and MBP-tagged IFT25 were used for *in vitro* binding assay. The left panel shows that immobilized GST-IFT27 protein on the beads could retain MBP-IFT25 protein but not the control protein, MBP. The right panel shows that immobilized MBP-IFT25 protein on beads could retain GST-IFT27 protein but not the control protein, GST. The molecular marker is labeled on the left of each figure. From top to bottom for both panels, the first figure is the Coomassie blue-stained gel. The second and third figures represent the immunoblots probed with antibodies against IFT25 and IFT27, respectively. The fourth one is the immunoblots probed with antibodies against either MBP (left panel) or GST (right panel). The loading materials for each lane of the gels are shown in the tables at the top of each panel. S stands for supernatant and P for bead pellet.

Previous studies have indicated that the human homologues of IFT25 and IFT27 interact directly in yeast two-hybrid analyses [34]. Based on the observation that *Chlamydomonas* IFT25 and IFT27 co-localized at regions around the transition zones, we questioned whether these two proteins also physically bind to each other. Herein, *in vitro* pull-down assays were applied (Fig. 5). As detailed in the materials and methods section, recombinant MBP-IFT25, GST and GST-IFT27 were expressed in bacteria and purified. Thereafter, MBP-IFT25 was mixed with beads bound with either GST-IFT27 or the control GST protein, while GST-IFT27 was mixed with beads coated with either MBP-IFT25 or the control protein MBP. The pull-down proteins were recovered by centrifugation and analyzed by immunoblotting. It was revealed that MBP-IFT25 but not MBP was recovered by the immobilized GST-IFT27, and GST-IFT27 but not GST was recovered by immobilized MBP-IFT25 (Fig. 5). Moreover, in a separate *in vitro* binding assay (Fig. S1), it was found that GST-IFT27 could be retained on amylose resins in the presence of MBP-IFT25 protein, and co-eluted from amylose resins with MBP-IFT25, but not with MBP. There was no co-elution observed either between MBP-IFT25 and GST or MBP and GST, indicating that the co-elution of IFT27 and IFT25 was specific and resulted from the direct interaction between IFT27 and IFT25. Based on these results, we concluded that IFT25 interacts with IFT27 directly.

### Preassembled IFT complex B contains substoichiometric levels of IFT25 and IFT27

**Figure 6:**
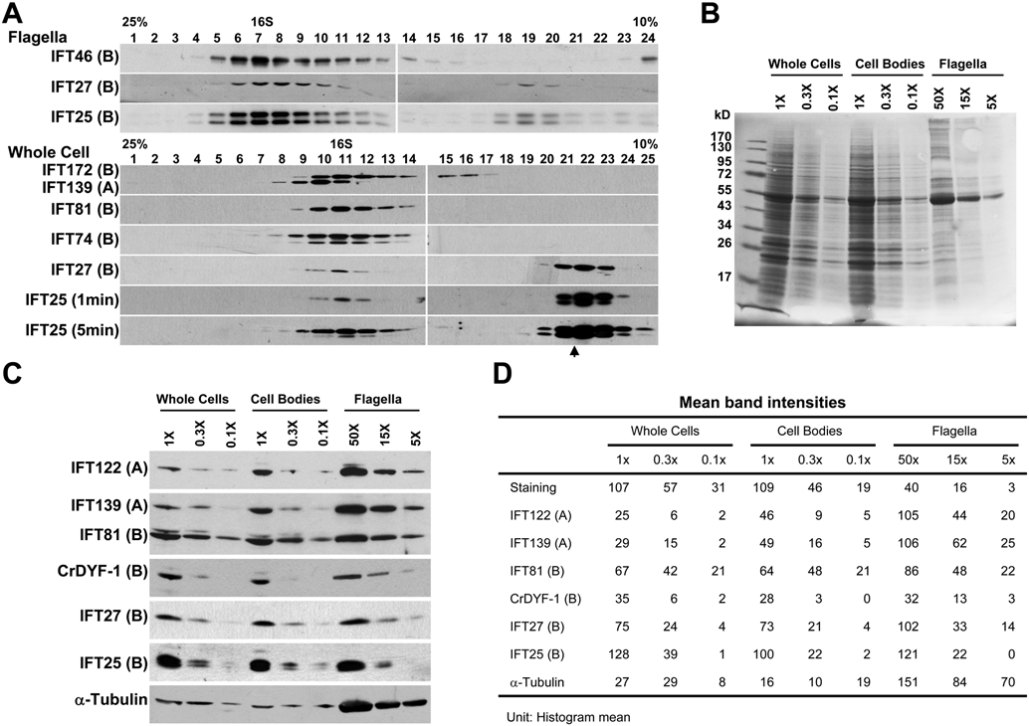
The majority of IFT25 and IFT27 are not integrated into the IFT complex B in the cell body. (A). Wild-type flagella extracts or cell body lysates of the *bld2* mutant were fractionated on a 10–25% sucrose gradient. The fractions were then separated by SDS-PAGE. Immunoblots probed with antibodies against several IFT particle proteins as indicated to the left of the blots. Flagellar IFT25 and IFT27 were found in two peaks, a major peak at 16 S fractions, and a minor peak at much lighter fractions. IFT46, a complex B subunit, only peaked at 16S fractions. Only a portion of whole-cell IFT25 and IFT27 was integrated into the preassembled IFT complex B in the cell body. IFT particle proteins, including complex A subunit IFT139 and complex B subunits IFT172, IFT81, and IFT74.were found entirely in 16S fractions. In contrast, IFT25 and IFT27 had two peaks on the gradient. The Western blots shown for IFT25 were exposed for 1 minutes or 5 minutes to ensure that phosphorylated IFT25 was detected in the 16S fractions. (B). Ponceau S staining of the membrane showing the protein profiles of whole cells, cell bodies, and flagella. Flagellar proteins were isolated from 5×10^6^ (5×), 1.5×10^7^ (15×), and 5×10^7^ (50×) cells. Whole cell or cell body proteins were isolated from 1×10^6^ (1×), 3×10^5^ (0.3×), and 1×10^5^ (0.1×) cells. (C). The distributions of IFT25 and IFT27 proteins among whole cells, cell bodies, and flagella. The same or a duplicated membrane shown in B was probed with antibodies as indicated to the left. (D). The intensity of each lane or band stained by Ponceau S or antibodies. Photoshop CS3 software was used for the measurement.

As shown above, IFT25 and IFT27 localized at regions around the transition zones, a localization pattern that has not been observed for other IFT particle subunits. This result indicated that partial IFT25 and IFT27 are not associated with other complex B subunits. It is known that deflagellation occurs at the distal ends of the transition zones, and both transition zones and basal bodies thus remain in the cell body rather than the flagella after deflagellation [40]. To investigate whether IFT25 and IFT27 are associated with complex B before they enter flagella, sucrose density gradient centrifugation was applied to extracts of the deflagellated cell bodies of wt cells or *bld2* mutant cells. *bld2* mutant was used to eliminate the influence of retrograde IFT on the assembly status of IFT particle proteins in the cell body, since it lacks flagella due to a mutation in the gene encoding ε-tubulin [41]. The result showed that in both wt (data not shown) and *bld2* cells, complex A subunit IFT139 and complex B subunits IFT172, IFT81, and IFT74 all peaked at the 16S fractions (Fig. 6A), indicating that complexes A and B are indeed preassembled in the cell body. It was the phosphorylated form of IFT25 that was preferentially incorporated into the IFT particle complexes (Fig. 6A), despite the fact that both phosphorylated and non-phosphorylated IFT25 were detected in the whole cell lysates (Fig. 6C). It was clear that IFT25 and IFT27 had a similar sedimentation pattern. Both were separated into two peaks, with one peak together with other complex A and complex B subunits at the 16S fractions, and another peak arose at much lighter fractions without the presence of other complex A and B subunits (Fig. 6A). This result showed that a portion of IFT25 and IFT27 were not associated with IFT particle complex B in the cell body. Unlike in the cell body, in the flagella, both phosphorylated and non-phosphorylated forms of IFT25 were found in the 16 S fractions (Fig. 6A); IFT25 and IFT27 were detected mainly in the 16S fractions on the sucrose density gradient (Fig. 6A), indicating that these two proteins are associated with IFT complex B in the flagella. On the sucrose gradients, the flagellar IFT25 and IFT27 were also consistently found to form a minor peak at much lighter fractions. This minor peak did not contain other IFT particle subunits, such as IFT46 (Fig. 6A). The significance of this minor peak for the function of IFT25 and IFT27 is unknown. Taken together, based on these results, we can deduce that the cell must either produce excessive IFT25 and IFT27 relative to other IFT particle proteins if the composition of IFT particle complex B is the same in the cytoplasm as in the flagella, or produce the same amounts of IFT25 and IFT27 relative to other IFT particle proteins while the preassembled IFT complex B in the cell body contains substoichiometric level of both proteins. These two possibilities are mutually exclusive.

Next, we measured the distribution ratio of the IFT proteins in the flagella versus the cell body after deflagellation (Figs. 6B, 6C, and 6D). The amounts of IFT25 and IFT27 isolated from the flagella of 1.5×10^7^ cells were about the same as that contained in the cell bodies of 3×10^5^ cells, indicating that only about 2% of these two proteins were distributed in the flagella. The distribution patterns of IFT25 and IFT27 were similar to complex B proteins IFT81 and CrDYF-1(an IFT complex B subunit, unpublished data). In contrast, about 20% of the total complex A subunits IFT122 and IFT139 were distributed to the flagella, since the amount of IFT139 or IFT122 in flagella isolated from 5×10^6^ cells was about equal to the amount contained in 1×10^6^ cell bodies (Fig. 6D). This result demonstrated that IFT complex B has a cell body pool size approximately ten times larger than IFT complex A. Based on this, it is clear that the cell does not produce excessive IFT25 and IFT27 relative to other IFT particle complex B proteins. Taken together with the results shown in Fig. 6A, it is concluded that the preassembled IFT complex B in the cell body must contain substoichiometric level of both proteins.

## Discussion

We report herein the identification of a new IFT particle protein of *Chlamydomonas*, IFT25. Using genetic, biochemical and immunofluorescence staining approaches, we have found that IFT25 fulfills all the criteria for an IFT particle protein. The evidence includes its cosedimentation with other IFT particle proteins in 16S fractions of the flagellar sucrose density gradients, its presence in flagella in a molar ratio relative to other IFT proteins (Fig. 1), its localization pattern in flagella as the punctual dots shown by other IFT particle proteins, and its dependence on Fla10-kinesin-II to enter flagella. By using both anterograde and retrograde IFT *ts* mutants as tools, we found that the amount change of IFT25 in flagella parallels with that of the IFT complex B subunits but not complex A subunits. Thus, IFT25 is an IFT complex B subunit.

Nucleotide sequence analysis showed that homologues of IFT25 and IFT27 are encoded in the genomes of many organisms that are able to assemble cilia and/or flagella, including humans, mice and zebrafish. Amino acid sequences of *Chlamydomonas* IFT25 and IFT27 are 37% and 38% identical to the corresponding human proteins. As expected, genes encoding the homologues of IFT25 and IFT27 are absent from the genomes of organisms that lack cilia and flagella. Interestingly, both proteins are also absent from the genomes of *D. melanogaster* and *C. elegans*, which utilize cilia for sensory signal detection. These observations suggest that IFT25 and IFT27 have specialized roles in IFT that are not required for ciliary assembly in the fly and worm, or their roles in ciliary assembly can be compensated by other proteins in these two organisms.

In this study, immunofluorescence staining assay showed that IFT25, similarly to IFT27 and other complex B subunits such as IFT46, has a punctuate flagella distribution as expected. Surprisingly, IFT25 shows a unique distribution pattern in the cell body. It is clear that IFT25 concentrates at regions around the transition zones as well as in basal bodies. IFT25 overlaps with IFT27, and their overlap region is shown as two bright spots directly above each basal body. As shown in Fig. 4, we cannot exclude the possibility that both proteins actually partially overlap with other complex B subunits such as IFT46 in the transition zones. In basal bodies, IFT25 alone is present at a region immediately below α-tubulin, and thus does not overlap or only partially overlaps with other IFT proteins such as IFT46. In addition, IFT25 interacts physically with IFT27. These observations together with the data showing that only a fraction of the cellular IFT25 and IFT27 are detected in the preassembled complex B in the cell body lead to a conclusion that IFT25 and IFT27 may exist as a subcomplex independent of complex B in the transition zones.

This study showed that the endogenous IFT27 resides solely at regions around the transition zones, a distribution pattern different from that observed previously with a C-terminal GFP-tagged IFT27 (IFT27-GFP) [33]. Although both IFT27 (Fig. 4) and IFT27-GFP [33] show a typical flagella distribution pattern for an IFT protein, IFT27-GFP only partially represents the true distribution pattern of the endogenous IFT27. This provides an explanation of why the function of IFT27-GFP is only a partial substitute for the function of the endogenous IFT27 in *Chlamydomonas*. This observation also indicates that even small tags, such as HA, may affect the expression pattern of the IFT particle proteins and their functions in ciliogenesis.

### Do the cellular pool sizes of complexes A and B have biological significance?

This study elucidated for the first time that the cytoplasmic precursor pool sizes of IFT particle complexes A and B are not the same: the amount of complex B is around ten times the amount of complex A (Fig. 6). In the flagella of wt cells, we know that equal numbers of complexes A and B are present in IFT particles [5]. At the first glance, it seems to be wasteful for the cell to produce a larger reservoir of complex B than complex A. However, the larger amount of prefabricated complex B might be beneficial to cells under certain conditions. For example, this study showed that in the flagella of the *fla15* mutant, the amount of complex B was dramatically increased while complex A disappeared almost completely (Fig. 3). The elevated amount of flagellar complex B might be essential to prevent IFT machinery from complete loss of function so that flagella can still be assembled. Furthermore, IFT complex B subunits are also involved in cell functions other than ciliogenesis. To date, at least two complex B subunits, IFT88 and IFT27, have been shown to participate in the regulation of cell cycle progression. IFT88 regulates G1-S phase transition in non-ciliated proliferating vertebrate cells [42], and IFT27, a Rab-like small GTPase, is an essential protein for normal cell cycle progression in *Chlamydomonas* [33].

### Are IFT25 and IFT27 together involved in the regulation of anterograde IFT?

One of the current outstanding questions in the field of cilia is how IFT is regulated. This study brought to light several clues that IFT25 and IFT27 play a role in the regulation of IFT at the flagellar base. In the cell body, the majority of IFT25 and IFT27 are not integrated into the preassembled complex B; other than those two subunits, all of the complex B components are assembled prior to their entrance into the flagella (Figs. 4 and 6). Considering that IFT25 (Fig. 1A and 6A) and IFT27 (Fig. 6A) [33] are integrated into IFT complex B again once they enter flagella, it is possible that both proteins are important factors in the regulation of IFT, most likely at the initiation stage. Furthermore, it is mainly the phosphorylated form of IFT25 that is integrated into the preassembled complex B, despite the fact that both phosphorylated and non-phosphorylated IFT25 were present in cell lysates (Fig. 6). In addition, instead of being concentrated in the basal body as described for other complex B subunits [16], IFT25 and IFT27 are co-localized at regions around the transition zones (Fig. 4). These observations hint that the initiation of anterograde IFT involves the assembly of IFT25 and IFT27 to the rest of the preassembled complex B at the flagellar base, and the phosphorylation state of IFT25 is critical for this process.

## Materials and Methods

### Strains and culture conditions

*Chlamydomonas reinhardtii* strains used in this study include: wt strain *137c* (CC-125); temperature-sensitive flagella assembly mutants *fla10-1* (CC-1919), *fla8* (CC-1396), *fla3-1b* [37], *fla15* (CC-3861), *fla16* (CC-3862), and *fla17-1* (CC-3863). Aside from *fla3-1b*, which was a gift from the Porter lab at the University of Minnesota, the rest of the strains were obtained from the *Chlamydomonas* center (http://www.chlamy.org). If not otherwise specified, cells were grown on Tris-acetate-phosphate (TAP) solid plates or in M1 liquid media with constant aeration in a Conviron programmed at 18°C with a light–dark cycle of 14:10 hours.

### Antibodies

Polyclonal α-IFT25 antisera were raised against an internal peptide of IFT25 protein, CDQDKPDFEKVFEVE. The subcutaneous injection of the peptide antigen into the rabbits for antisera generation and the subsequent affinity purification were performed by Pocono Rabbit Farm & Laboratory Inc. (Canadensis, PA).

Other antibodies used in this study include antibodies against α-tubulin (clone B-5-1-2, ascites fluid; Sigma); IC69 (clone 1869A; Sigma); acetylated tubulin (clone 6-11B-1, ascites fluid; sigma); MBP (New England biolabs), and GST (clone 9AT106, ABGENT). IFT polypeptide antibodies include antibodies to *C. reinhardtii* IFT172, IFT81, IFT139 [5], IFT46 [43], CrDYF-1 (Qin, unpublished), and IFT27 (the antibody raised against the MBP tagged full length IFT27, then affinity-purified by using the GST-tagged full-length IFT27 protein, refers to [33]); FLA10 [5] and KAP [37]; and D1bLIC [11]. The antibody against IFT122 was a gift from Dr. Cole at the University of Idaho.

### Preparation of cell body and flagella extracts

Flagella isolation was performed as previously described [5], [14], [43] with modifications to improve the yield of IFT particle proteins. Briefly, 32 liters of *CC-125* cells grown to log phase in TAP medium were collected and resuspended in 4 liters of 10 mM HEPES, pH 7.2. Cells were aerated under light for 1–2 hours to ensure the flagellation of cells. Thereafter, cells were concentrated in 400 ml of 10 mM HEPES, pH 7.2 and incubated with 5% sucrose under constant stirring for 30 minutes. After deflagellation by pH shock, the mixture of detached flagella and cell bodies were underlaid with 25% sucrose and centrifuged to remove the cell bodies in conical tubes in a swinging bucket rotor. This step was repeated once to reduce the contamination of cell bodies in the isolated flagella. Flagella in the supernatants were collected by centrifugation at 10,000 rpm for 10 minutes in a Beckman Coulter JA-20 rotor. The pellet was suspended in 300–500 µl of 1×HMDEK buffer (10 mM HEPES pH 7.4, 5 mM MgSO_4_, 1 mM DTT, 0.5 mM EGTA and 25 mM KCl) plus protease inhibitors aprotinin (2 µg/ml), pepstatin (1 µg/ml) and PMSF (1 mM). The axonemes were removed from the flagella extracts by two cycles of freeze (−80°C) and thaw followed by centrifugation.

Flagella extracts shown in Figs. 2 and 3 were isolated from 16 liters of mutants as described above, but without incubation with 5% sucrose before deflagellation. The cells were then incubated in a 32°C water-bath for 0, 1.0, or 1.5 hours. Cell body extracts used in Fig. 6 were prepared by employing mechanical force to disrupt cell membranes as described elsewhere [14], [43]. The percentage of IFT25 distributed to the flagella versus the cell body was determined as described previously [44]. Briefly, *CC-125* cells grown to log phase were collected and the cell density was determined with a hemacytometer. After deflagellation by pH shock, both flagella and cell bodies were collected by centrifugation. Flagella in the supernatant fraction were harvested by centrifugation at 14,000 rpm (Eppendorf, 5415R) for 10 minutes at 4°C.

### Sucrose density gradients

Linear 12 ml 10–25% sucrose density gradients in 1×HMDEK buffer plus protease inhibitors were generated by using the Jule Gradient Former (Jule, Inc. Milford) and used within 1 hour. No more than 700 µl of cell body or flagella extracts were loaded on the top of the gradients and separated at 38,000 rpm, 4°C, for 14 hours in a SW41Ti rotor (Beckman Coulter). The gradients were fractioned into 24 to 26 0.5 ml aliquots by using a Pharmacia LKB Pump P-1 coupled with a FRAC-100 fraction collector. The standards used to calculate S-values were BSA (4.4S), aldolase (7.35S), catalase (11.3S), and thyroglobulin (19.4S).

### SDS-PAGE and immunoblotting

Protein samples were separated by 10 % SDS-PAGE, and then electrotransferred onto a nitrocellulose membrane (Protran BA83, 0.2 µm, Whatman, Dassel, Germany). Before immunoblotting, the membrane was stained with Ponceau S to ensure that proteins were properly transferred. Thereafter, the membrane was blocked with 5% non-fat dry milk in TBS (10 mM Tris, pH 7.5, 166 mM NaCl) plus 0.05%Tween-20. Primary antibodies were diluted in the blocking solution and then incubated with the membrane overnight at 4°C. After washing three times with TBS plus 0.05% Tween-20, horseradish peroxidase-conjugated secondary antibodies (Pierce Biotechnology, Rockford, IL)) and chemiluminescence were used to detect the primary antibodies.

### Fluorescent microscopy

Immunofluorescence staining assay was performed as described elsewhere [38] with some modifications. The experiments were performed at room temperature if not otherwise specified. Wild-type cells grown under continuous light were seeded to 0.1% polyethyleneimine-coated coverslips for 8 minutes under strong light. The cells were permeablized and fixed with methanol twice each for 10 minutes at −20°C. Thereafter, cells were rehydrated with phosphate buffered saline (PBS) and incubated overnight in blocking buffer (5% BSA, 1% cold water fish gelatin, and 10% goat serum in PBS) at 4°C. The next day, cells were incubated with primary antibodies (1° Abs) for 4 hours. After washing ten times in PBS, cells were incubated with secondary antibodies (2° Abs, 1:1000 dilution) for 2 hours. After washing the cells an additional ten times in PBS, the coverslips were mounted with SlowFade Antifade reagent (Molecular Probe, Eugene, OR) and cells were viewed on an Olympus IX-70 inverted fluorescent microscope at 150× amplification. Images were captured with a CoolSNAP HQ^2^ CCD camera (Photometrics, Tucson, AZ) with an exposure time between 0.2 and 1.5 seconds. The images were processed with the PCI software package (Hamamatsu Corporation, Bridgewanter, NJ) and ImageJ (free software from NCBI). The 1° Abs against IFT25 (1:10 dilution), IFT27 (1:10 dilution), IFT46 (1:1000 dilution), and α-tubulin (1:1000 dilution) have been described in the antibodies section above. The 2° Abs were Alexa-Fluor594 conjugated goat anti-rabbit or Alexa-Flour488 conjugated goat anti-mouse IgG (Molecular Probes, Eugene, OR). The dilution of antibodies was performed in blocking solution.

### *In vitro* binding assay

Two strains of *E.coli* BL21 (DE3) pLysE that harbor expression vectors, one with pGEX-2T-IFT27 and the other with pMAL-c2-IFT25, were utilized to express GST-IFT27 and MBP-IFT25 fusion proteins, respectively. GST protein control was produced in *E. coli* BL21 (DE3) pLysE harboring pGEX5X-2T vector (Pharmacia). MBP protein was either purchased from New England Biolabs (Beverly, MA) (used in Fig. 5) or produced in *E. coli* harboring pMAL-c2 vector (New England Biolabs) (used in Fig. S1). The details of vector construction and protein expression are available upon request. The results shown in Fig. 5 were obtained by following the procedures described below. Cell lysates collected from 1 to 2 liters of the cell cultures were incubated with 600 µl of 50% GST Glutathione Sepharose 4B (GE Healthcare) for GST and GST-IFT27 or Amylose Resin (New England Biolabs) for MBP-IFT25 for 1 hour. The beads were recovered by centrifugation at 5,000 rpm for 5 minutes and washed 5 times with 1×HMDEK. GST-IFT27 and MBP-IFT25 proteins were eluted from beads with 10 mM reduced glutathione and 10 mM maltose in 1×HMDEK, respectively. Beads bound with GST-IFT27 (∼2 µg) were incubated with MBP-IFT25 (∼2 µg), or MBP-IFT25 (∼2 µg) plus MBP (2 µg), or MBP (2 µg) for 1 hour. In a parallel set of experiments, beads bound with MBP-IFT25 (∼2 µg) were incubated with purified GST-IFT27 (∼2 µg), or GST-IFT27 (∼2 µg) plus GST (∼2 µg) for 1 hour. The supernatants and pellets were separated by low-speed centrifugation. The pellets were washed 4 times in 1×HMDEK, and then resuspended in 1×HMDEK at a 1:1 ratio to their corresponding supernatants. The bound proteins on the beads and their supernatants were boiled for 3 minutes in Laemmli protein gel loading buffer, then 15 µl of each sample was applied to SDS-PAGE and immunoblotting analysis. All the above steps were performed at 4°C if not otherwise specified.

## Supporting Information

Figure S1 Direct interaction between IFT25 and IFT27. Bacterial expressed MBP-IFT25, GST-IFT27, MBP, and GST were purified for in vitro binding assays. Individual purified protein was dissolved in 20 mM Tris pH7.4, 50 mM NaCl, 5 mM MgCl_2_ at a final concentration of 5 µM. Four parallel binding assays were then carried out simultaneously. Within each binding assay, two input proteins as indicated above the stained SDS-PAGE gel were incubated with 30 µl amylose resins (New England Biolabs Inc) for 1 hour. The supernatants were then removed by low speed centrifugation. The remaining beads were washed 10 times by 0.5 ml washing buffer (20 mM Tris pH7.4, 50 mM NaCl, 5 mM MgCl_2_); and subsequently incubated with 100 µl elution buffer (10 mM maltose in washing buffer). The eluted supernatants (marked as “E” above the stained gel) were collected by low speed centrifugation. Finally, 10 µl of the input samples (indicated as “I” above the PAGE gel) and their corresponding eluted supernatants were applied to 10% SDS-PAGE. The proteins on the gel were visualized by Coomassie blue staining. All the above procedures were performed at room temperature. The result showed that GST-IFT27 was co-eluted with MBP-IFT25 from amylose resins, but not with MBP. Co-elution was not observed either between MBP-IFT25 and GST or MBP and GST. “*” was used to mark a nonspecific protein co-purified with MBP-IFT25.
(2.85 MB TIF)
