# Supplementary figures and images for: Intraflagellar Transport (IFT) Protein IFT25 Is a Phosphoprotein Component of IFT Complex B and Physically Interacts with IFT27 in Chlamydomonas
Source: PLoS One. 2009 May 1;4(5):e5384. doi: 10.1371/journal.pone.0005384 (PMC2671599; doi:10.1371/journal.pone.0005384)

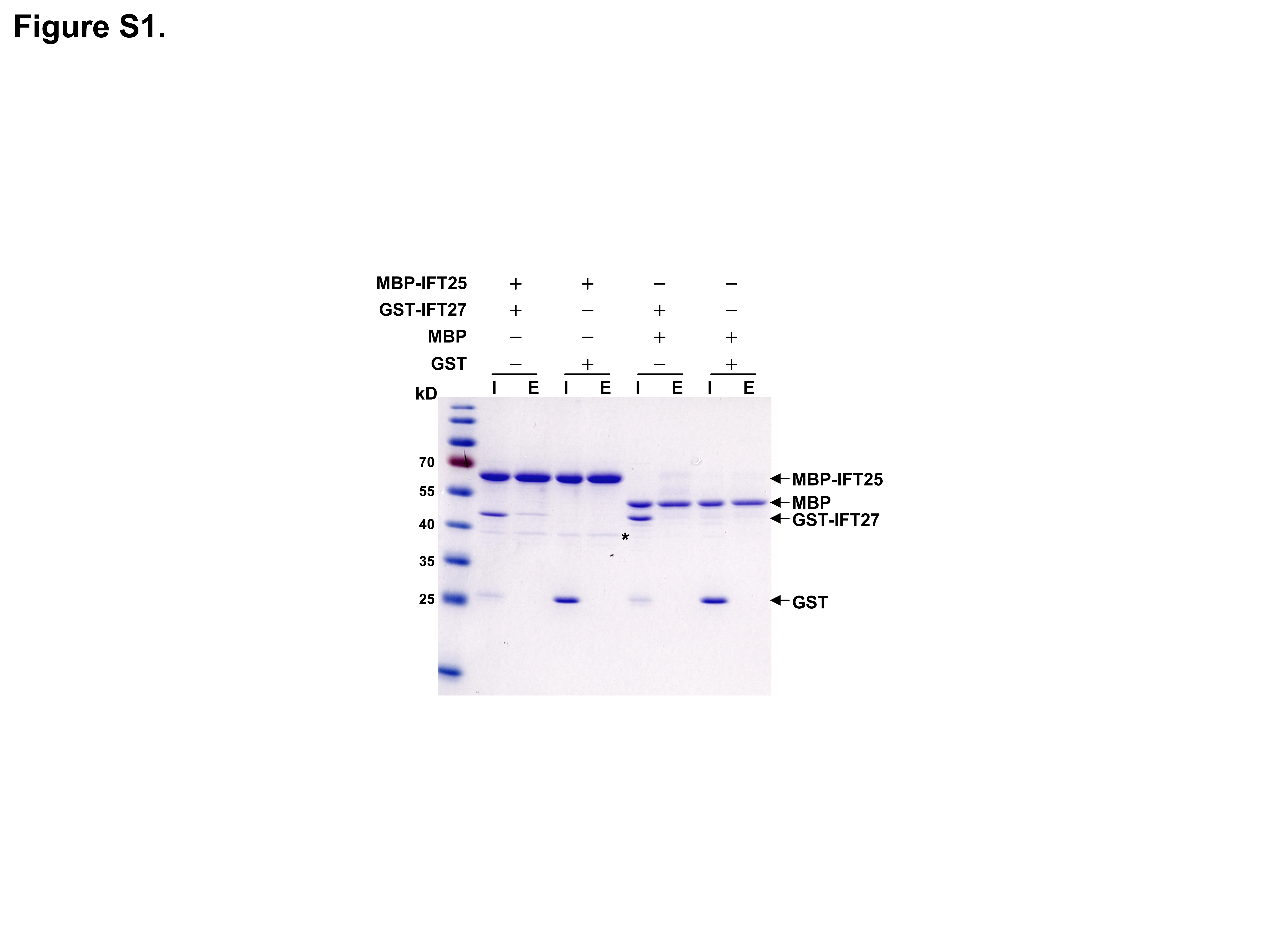

Supplement: Figure S1 — Direct interaction between IFT25 and IFT27. Bacterial expressed MBP-IFT25, GST-IFT27, MBP, and GST were purified for in vitro binding assays. Individual purified protein was dissolved in 20 mM Tris pH7.4, 50 mM NaCl, 5 mM MgCl2 at a final concentration of 5 µM. Four parallel binding assays were then carried out simultaneously. Within each binding assay, two input proteins as indicated above the stained SDS-PAGE gel were incubated with 30 µl amylose resins (New England Biolabs Inc) for 1 hour. The supernatants were then removed by low speed centrifugation. The remaining beads were washed 10 times by 0.5 ml washing buffer (20 mM Tris pH7.4, 50 mM NaCl, 5 mM MgCl2); and subsequently incubated with 100 µl elution buffer (10 mM maltose in washing buffer). The eluted supernatants (marked as “E” above the stained gel) were collected by low speed centrifugation. Finally, 10 µl of the input samples (indicated as “I” above the PAGE gel) and their corresponding eluted supernatants were applied to 10% SDS-PAGE. The proteins on the gel were visualized by Coomassie blue staining. All the above procedures were performed at room temperature. The result showed that GST-IFT27 was co-eluted with MBP-IFT25 from amylose resins, but not with MBP. Co-elution was not observed either between MBP-IFT25 and GST or MBP and GST. “*” was used to mark a nonspecific protein co-purified with MBP-IFT25. (2.85 MB TIF) [file pone.0005384.s001.tif]
